# miRNAs Caught Up in Metabolic Organ Crosstalk to Combat Obesity

**DOI:** 10.1016/j.ebiom.2016.03.007

**Published:** 2016-03-08

**Authors:** James M. Ntambi

**Affiliations:** Department of Biochemistry, University of Wisconsin-Madison, 433 Babcock Drive Madison, WI 53706, USA

Obesity has reached epidemic proportions globally as a public health problem and is a major contributor to the development of metabolic disease, including type II diabetes, coronary heart disease, hypertension, osteoarthritis, fatty liver disease, inflammation, sleep apnea and certain cancers. Currently there are no effective drugs for the treatment of obesity.

Obesity is a multifactorial condition stemming from a combination of genetic, dietary, and environmental factors and the interaction between these components. The precise etiology of the many abnormalities that occur in obesity is still unknown; however, an increasing body of evidence supports the Barker hypothesis that their true origins may actually occur *in utero* (Barker et al. 1990). The most widely accepted mechanism is that of fetal programming by nutritional stimuli due to changes in epigenetic modification of gene expression.

There are also many studies that show that several manifestations of the metabolic syndrome are associated with alterations in intracellular lipid and carbohydrate metabolism (Burhans and Ntambi 2016). It is generally undisputed that excess adipose tissue increases one's risk for the development of metabolic dysfunction. More specifically, visceral adiposity is now recognized as one of the strongest predictors of ectopic fat accumulation in tissues other than in adipose, such as in the liver and muscle.

The mechanisms that connect increased adiposity to the development of other metabolic diseases continue to be actively investigated. Perhaps unsurprisingly, much attention has focused on the role of secreted molecules that regulate metabolism systemically. While many of these secreted factors are peptides with well described target tissues, receptor partners and signaling cascades, recently there has also been an emergence of reports of non-protein factors, including specific lipids and fatty acids that control metabolic processes (Cao et al., 2008, Liu et al., 2013).

More recently, micro RNAs (miRNAs), small non-coding RNA molecules containing about 22 nucleotides that are found in many organisms and mainly function in the regulation of gene expression have emerged as key regulators of metabolism (Liu et al. 2014). In the current issue of *EBioMedicine*, Zhang et al., 2016-in this issue present a potentially interesting study investigating the role of a specific miRNA, miR-378, in metabolic regulation.

In the present work, Zhang et al., 2016-in this issue describe a complex hyper metabolic phenotype arising from whole body overexpression of miR-378 in mice. miR-378 can apparently prevent and treat obesity by activating the muscle pyruvate-phosphoenolpyruvate (pyruvate-PEP) futile cycle by targeting the Akt1-Foxo1-PEPCK pathway and enhancing lipolysis in adipose tissue. Activation of the futile cycle by miR-378 leads to impairment of glucose metabolism because, under these conditions, the pyruvate from glycolysis does not enter the tricarboxylic acid (TCA) cycle. Instead, it is converted back into PEP in a gluconeogenic reaction catalyzed by phosphoenolpyruvate carboxykinase (PEPCK) and pyruvate kinase (PC), which costs ATP and produces an energy deficiency. As consequence more energy from adipose tissue lipolysis is needed to balance energy homeostasis.

Zhang et al., 2016-in this issue attribute the elevated lipolysis to the miRNA-378-mediated decrease in expression of stearoyl-CoA desaturase-1 (SCD1) in adipose tissue. SCD1 catalyzes the synthesis of monounsaturated fatty acids from saturated fatty acids, mainly palmitate and stearate into palmitoleate and oleate, respectively (Ntambi 1995). Global knockout of SCD1 in mice displays a similar phenotype to that of miR-378 transgenic mice, including a lean body phenotype, elevated lipolysis and increased energy expenditure (Sampath and Ntambi 2014). However, adipose tissue specific knockout of SCD1 does not affect lipolysis, (Flowers et al., 2012) suggesting that miR-378 may be targeting SCD1 expression in other tissues. Alternately, other mechanisms involved in miR-378-mediated adipocyte lipolysis that have been reported (Kulyte et al. 2014) could be responsible. Further, the role of miR-378 in regulating molecules that promote lipolysis, including adrenaline, glucagon, thyroid hormone, adipokines, and hepatokines, that signal energy insufficiency and mediate inter-organ cross talk could also contribute to the mechanism of miR-378 in maintaining whole body energy homeostasis. It will also be interesting to determine whether miR-378 overexpression causes an inflammatory response, which is known to induce lipolysis in adipose tissue.

In general, the data by Zhang et al., 2016-in this issue suggest that the regulation of futile cycles is important in maintaining whole body metabolic homeostasis. Futile cycles are typically regarded as energetically wasteful processes that are avoided in metabolic pathways. Not so any more. The miR-378-activated pyruvate-phosphoenolpyruvate futile cycle is the primary cause of elevated lipolysis in adipose tissues and helps orchestrate inter-organ crosstalk between skeletal muscle and fat to control systemic energy homeostasis in mice. Thus, miR-378 could be implicated in preventing and treating obesity in humans.

## Conflicts of Interest

The author declares no conflicts of disclosure.
